# MicroRNA-93 promotes the malignant phenotypes of human glioma cells and induces their chemoresistance to temozolomide

**DOI:** 10.1242/bio.015552

**Published:** 2016-05-16

**Authors:** Rui Chen, Huan Liu, Quan Cheng, Bing Jiang, Renjun Peng, Qin Zou, Wenren Yang, Xiaosheng Yang, Xiaobing Wu, Zigui Chen

**Affiliations:** 1 Department of Neurosurgery, Nanhua Hospital Affiliated to Nanhua University, Hengyang, Hunan 421001, China; 2 Department of Cardiology, Nanhua Hospital Affiliated to Nanhua University, Hengyang, Hunan 421001, China; 3 Department of Neurosurgery, Xiangya Hospital of Central South University, Changsha, Hunan 410008, China

**Keywords:** Glioma, MicroRNA, Proliferation, Invasion, Cell cycle, Temozolomide

## Abstract

MicroRNAs (miRNAs), a class of small non-coding RNAs, can induce mRNA degradation or repress translation by binding to the 3′-untranslated region (UTR) of its target mRNA. Recently, some specific miRNAs, e.g. miR-93, have been found to be involved in pathological processes by targeting some oncogenes or tumor suppressors in glioma. However, the regulatory mechanism of miR-93 in the biological behaviors and chemoresistance of glioma cells remains unclear. In the present study, *in situ* hybridization and real-time RT-PCR data indicated that miR-93 was significantly upregulated in glioma patients (*n*=43) compared with normal brain tissues (*n*=8). Moreover, the upregulated miR-93 level was significantly associated with the advanced malignancy. We also found that upregulation of miR-93 promoted the proliferation, migration and invasion of glioma cells, and that miR-93 was involved in the regulation of cell cycle progression by mediating the protein levels of P21, P27, P53 and Cyclin D1. P21 was further identified as a direct target of miR-93. Knockdown of P21 attenuated the suppressive effects of miR-93 inhibition on cell cycle progression and colony formation. In addition, inhibition of miR-93 enhanced the chemosensitization of glioma cells to temozolomide (TMZ). Based on these above data, our study demonstrates that miR-93, upregulated in glioma, promotes the proliferation, cell cycle progression, migration and invasion of human glioma cells and suppresses their chemosensitivity to TMZ. Therefore, miR-93 may become a promising diagnostic marker and therapeutic target for glioma.

## Abstract (precis)

**Summary:** MiR-93, an oncogene in glioma, is able to confer glioma cells chemoresistance to temozolomide, which may become a promising therapeutic target for glioma.

## INTRODUCTION

Glioma is the most common cancer in central nervous system, accounting for about 80% of malignant tumors in brain (Siegel et al., 2015b). Advanced glioma shows rapid growth and strong invasiveness, with high mortality and recurrence rates after surgical resection (Torre et al., 2015). Although improvements have been made in the combined therapies including surgical resection, chemotherapy and radiotherapy, the prognosis of glioblastoma patients still remains poor, with a median survival time of only 12 months (Siegel et al., 2015a). Therefore, it is urgent to explore the molecular mechanism involved in glioma for the development of effective therapeutic strategies (Auffinger et al., 2013).

MicroRNAs (miRNAs), a class of small non-coding RNAs with 18-25 ribonucleotides, can induce mRNA degradation or act as repressors of translation through directly binding to the 3′-untranslated region (UTR) of their target mRNA (Petri et al., 2014). Through negatively mediating the protein levels of their target genes, miRNAs have been implicated in various biological processes, such as cell proliferation, apoptosis, cell cycle progression, differentiation, autophagy, migration, metabolism, and so forth (Wei et al., 2013). Moreover, as their target genes include many oncogenes and tumor suppressors, miRNAs also play key roles in the development and progression of human cancers (Areeb et al., 2015). Some specific miRNAs have been reported to be upregulated or downregulated in glioma samples, and function as oncogenes or tumor suppressors, involved in the regulation of glioma cell survival, proliferation, cell cycle progression, migration, invasion, angiogenesis, as well as chemotherapy or radiotherapy resistance (Nikaki et al., 2012). For instance, miR-873 was found to enhance the sensitivity of glioma cells to cisplatin by targeting Bcl-2 (Chen et al., 2015). MiR-146b was reported to suppress glioma cell proliferation and induce apoptosis by targeting TRAF6, and predict the prognosis of glioma patients (Liu et al., 2015).

MiR-93 has been demonstrated to play a key role in the development, progression and chemotherapy resistance in several common human cancers. Ohta et al. reported miR-93 could enhance the proliferation, migration and invasion, while inhibited the apoptosis in hepatocellular carcinoma cells through directly targeting PTEN and CDKN1A and activating the c-Met/PI3K/Akt pathway (Ohta et al., 2015). MiR-93 was also found to promote the proliferation, invasion and metastasis of nasopharyngeal carcinoma cells *in vitro* and *in vivo* by directly targeting TGFbetaR2 (Lyu et al., 2014). Recently, miR-93 was found to promote tumor growth and angiogenesis by targeting integrin-β8 and activation of PI3K/Akt signaling pathway (Fang et al., 2011; Jiang et al., 2015). Besides, miR-93 was also suggested to be involved in glioma immune escape (Codo et al., 2014). However, the detailed regulatory mechanism of miR-93 in glioma remains still largely unclear. Therefore, our study aimed to explore the expression and function of miR-93 in the regulation of the malignant phenotypes of glioma cells, as well as the underlying mechanism.

## RESULTS

### MiR-93 is upregulated in glioma tissues compared to normal brain tissues

**Fig. 1.**
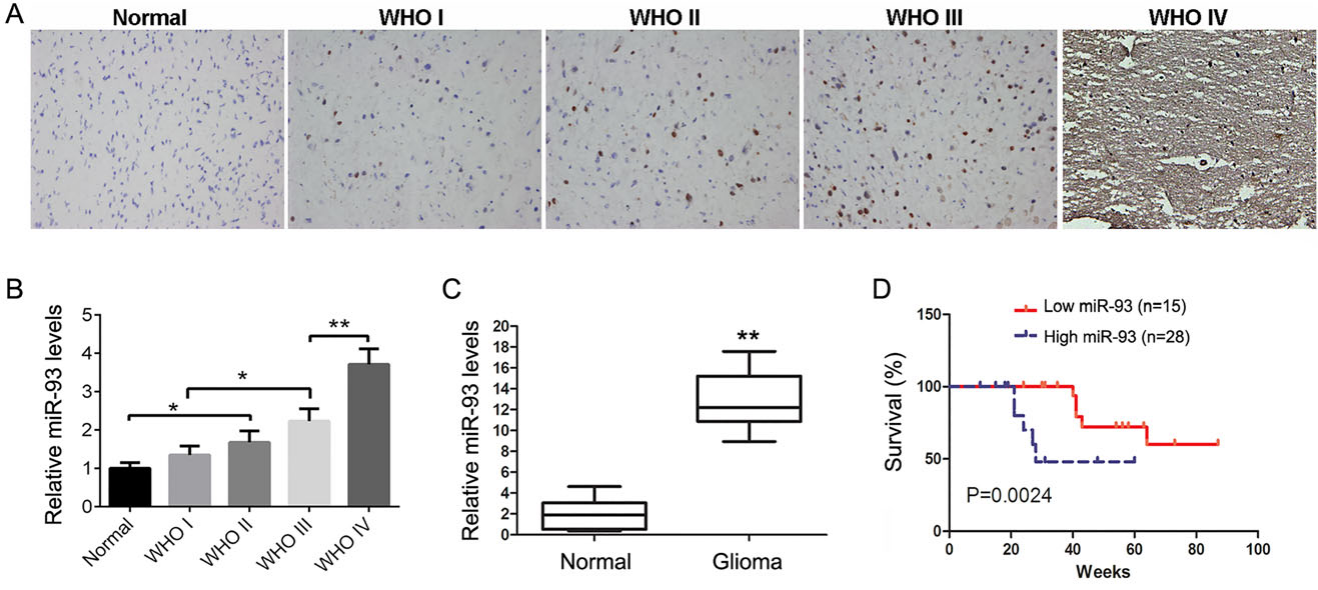
**The expression of miR-93 in glioma.** (A) Representative images of *in situ* hybridization staining in glioma tissues. Magnification, 200×. (B) Relative score of miR-93 expression in normal brain tissues and in different grade glioma tissues, indicating that the miR-93 level was gradually upregulated as the advanced malignancy of glioma. Data represented as mean±s.d; *P<0.05, **P<0.01. (C) Real-time RT-PCR data showed that miR-93 was significantly upregulated in glioma tissues compared to normal brain tissues. **P<0.01. (D) Kaplan–Meier curves showed worse overall survival rates for glioma patients with high miR-93 expression (*n*=28) compared to patients with low miR-93 expression (*n*=15) (*P*=0.0024).

To reveal the role of miR-93 in glioma, we firstly examined the expression levels of miR-93 in 43 cases of glioma tissues as well as eight cases of normal brain tissues by conducting in-site hybridization and real-time RT-PCR. *In situ* hybridization data showed that miR-93 was positively expressed in 38 cases of glioma tissues, and the positive expression rate was 88.4% (38/43). However, the positive expression rate of miR-93 in normal brain tissues was only 25% (2/8), significantly lower than that in glioma tissues (*P*<0.01). In addition, *in situ* hybridization data also indicated that miR-93 was gradually upregulated as the malignant progression of glioma (Fig. 1A,B). Real-time RT-PCR also showed similar findings that miR-93 was significantly upregulated in glioma tissues compared to normal brain tissues (Fig. 1C).

### High miR-93 level is associated with the advanced malignancy and poor prognosis of glioma patients

We further analyze the relationship between the miR-93 levels and the clinicopathological features of glioma. The expression levels of miR-93 were positively correlated to the glioma grade (*P*<0.01). In addition, Kaplan–Meier survival time analysis showed that higher miR-93 levels were significantly associated with shorter survival time of patients with astrocytoma, indicating that its expression level is associated with the worse prognosis (Fig. 1D).

### MiR-93 promotes the malignant phenotypes of glioma cells

**Fig. 2.**
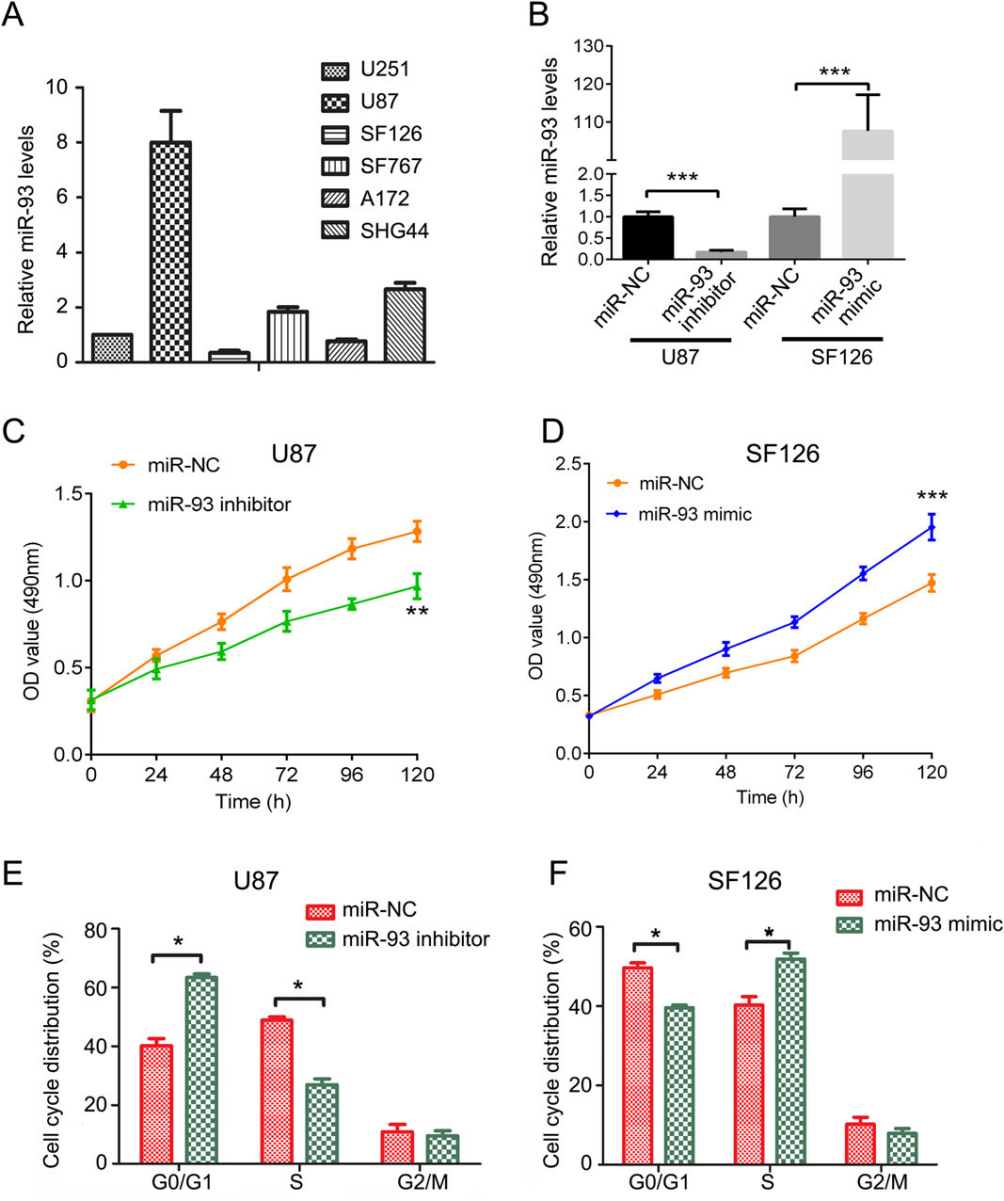
**Downregulation of miR-93 inhibits cell proliferation and arrests cell cycle in U87 and SF126 cells.** (A,B) Real-time RT-PCR was performed to analyze the miR-93 levels in several glioma cell lines including U87, U251, SF126, SF767, A172 and SHG44 (A), and in U87 and SF126 cells transfected with miR-93 inhibitor or mimic, respectively (B). Cells transfected with scramble miRNA (miR-NC) were used as control. (C,D) MTT assay was performed to determine the cell proliferation in U87 cells (C) and SF126 cells (D) after miR-93 inhibitor or mimic transfection. (E,F) Cell cycle analysis was performed to examine the cell cycle distribution in U87 cells (E) and SF126 cells (F) after miR-93 inhibitor or mimic transfection. Data represented as mean±s.d; *P<0.05, **P<0.01, ***P<0.001 vs miR-NC.

*In vitro* study was further performed to investigate the detailed role of miR-93 in glioma. Its expression levels were firstly examined in several common glioma cell lines including U87, U251, SF126, SF767, A172 and SHG44 by conducting real-time RT-PCR. As indicated in Fig. 2A, U87 cells showed the highest miR-93 levels, while SF126 cells showed the lowest miR-93 levels. Therefore, we used U87 and SF126 cell lines in the following experiments. To knockdown the miR-93 levels in U87 cells, they were transfected with inhibitor. As demonstrated in Fig. 2B, transfection with miR-93 inhibitor led to a significant decrease in the mir-93 levels in U87 cells, when compared to the non-transfected U87 cells. To upregulate the miR-93 levels in SF126 cells, miR-93 mimic was used. Transfection with miR-93 mimic significantly enhanced the miR-93 levels in SF126 cells compared to control group. MTT assay was further conducted to examine cell proliferation. We observed that inhibition of miR-93 expression caused a significantly reduction in U87 cell proliferation, while overexpression of miR-93 markedly promoted SF126 cell proliferation (Fig. 2C,D). We further examined the cell cycle distribution. Knockdown of miR-93 in U87 cells significantly induced a cell cycle arrest at G0/G1 stage (Fig. 2E), while overexpression of miR-93 promoted the cell cycle progression in SF126 cells (Fig. 2F). These findings suggest that miR-93 plays an oncogenic role in the growth of glioma probably via promoting the cell cycle progression.

**Fig. 3.**
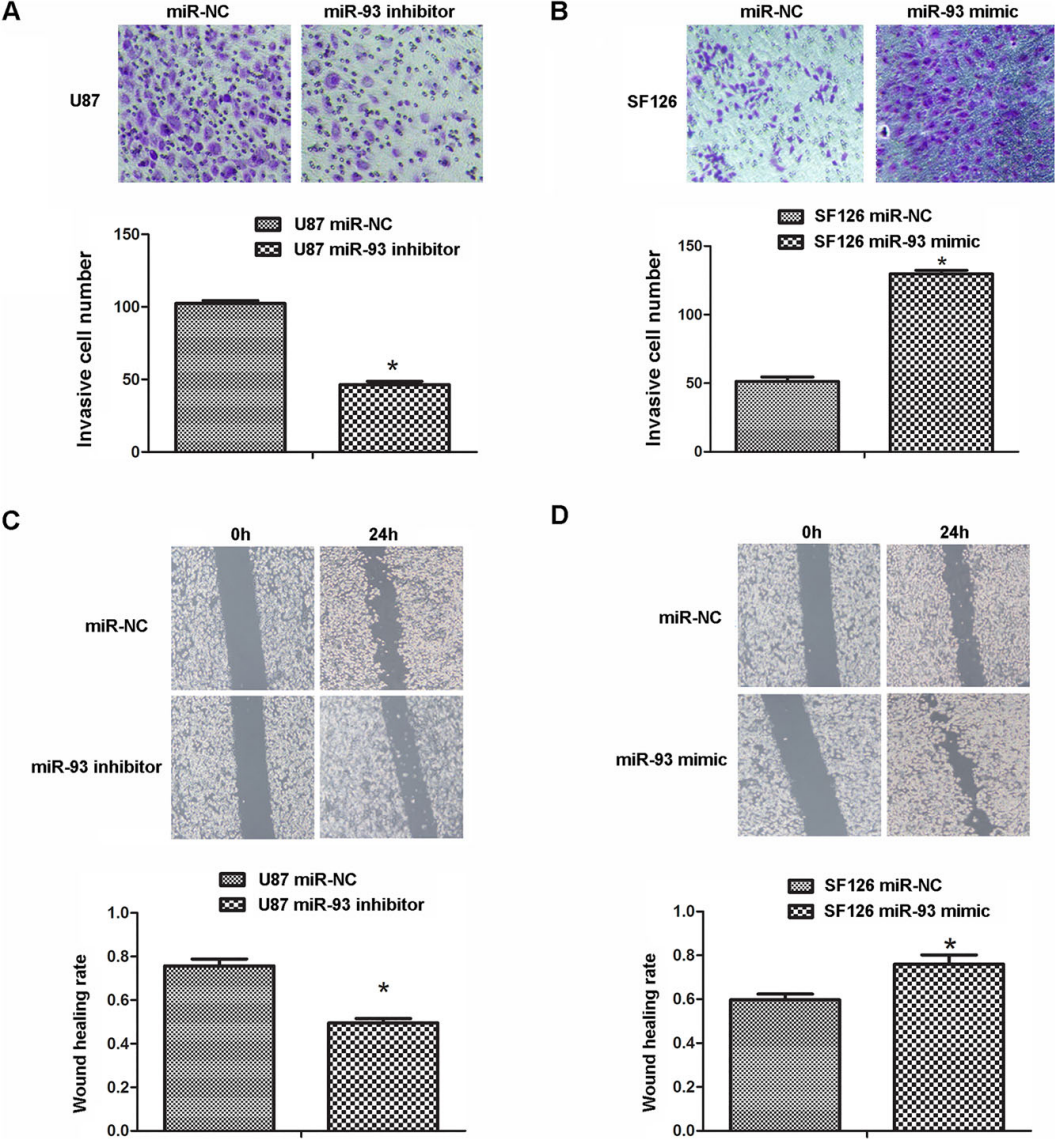
**Downregulation of miR-93 inhibits cell invasion in U87 and SF126 cells.** U87 and SF126 cells were transfected with miR-93 inhibitor or mimic, respectively. Cells transfected with scramble miRNA (miR-NC) were used as control. (A,B) Transwell assay was conducted to examine the cell invasion in U87 cells (A) and SF126 cells (B). (C,D) Wound healing assay was performed to determine the cell migration in U87 cells (C) and SF126 cells (D). Data represented as mean±s.d; *P<0.05 vs miR-NC.

We further studied the effects of miR-93 overexpression or inhibition on the invasion and migration of glioma cells by conducting transwell assay and wound healing assay. As indicated in Fig. 3A and B, inhibition of miR-93 significantly suppressed U87 cell invasion, while upregulation of miR-93 enhanced SF126 cell invasion. Similarly, knockdown of miR-93 decreased U87 cell migration, while overexpression of miR-93 promoted SF126 cell migration, compared to the control group, respectively. Accordingly, we suggest that miR-93 may play a promoting role in glioma metastasis.

### P21, a direct target of miR-93, is involved in miR-93-mediated glioma cell proliferation

**Fig. 4.**
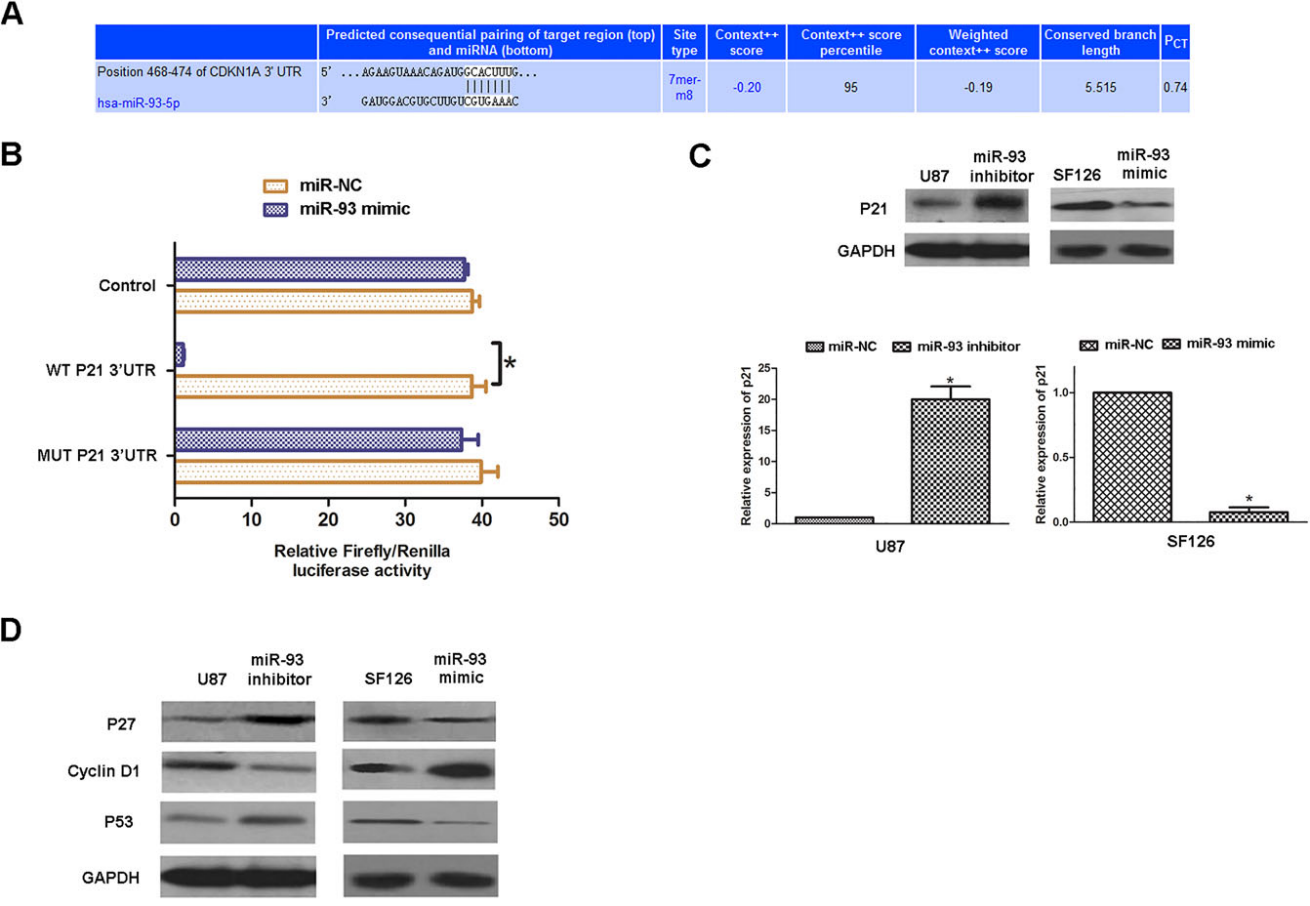
**MiR-93 directly targets p21.** (A) Targetscan software data indicated that CDKN1A (P21) was a potential target of miR-93. (B) We constructed the wild-type (WT) P21 3′UTR containing the putative binding sequences (GCACUUU) of miR-93 and the mutant type (MUT) P21 3′UTR with the binding sequences were changed into ‘AAAAAAA’. We then inserted them into the pMIR- REPORT miRNA Expression Reporter vector, generating WT P21-3′UTR plasmid and MUT P21-3′UTR plasmid, respectively. HEK293 cells were co-transfected with WT P21-3′UTR or MUT P21-3′UTR plasmid, and miR-NC or miR-93 mimic, respectively. After co-transfection for 48 h, dual-luciferase reporter assay was performed to examine the luciferase activity in each group. *P<0.05. (C,D) U87 and SF126 cells were transfected with miR-93 inhibitor or mimic, respectively. Cells transfected with scramble miRNA (miR-NC) were used as control. Western blot was conducted to examine the protein expression of P21 (C), P27, P53 and Cyclin D1 (D) in each group. GAPDH was used as an internal reference. Data represented as mean±s.d; *P<0.05.

**Fig. 5.**
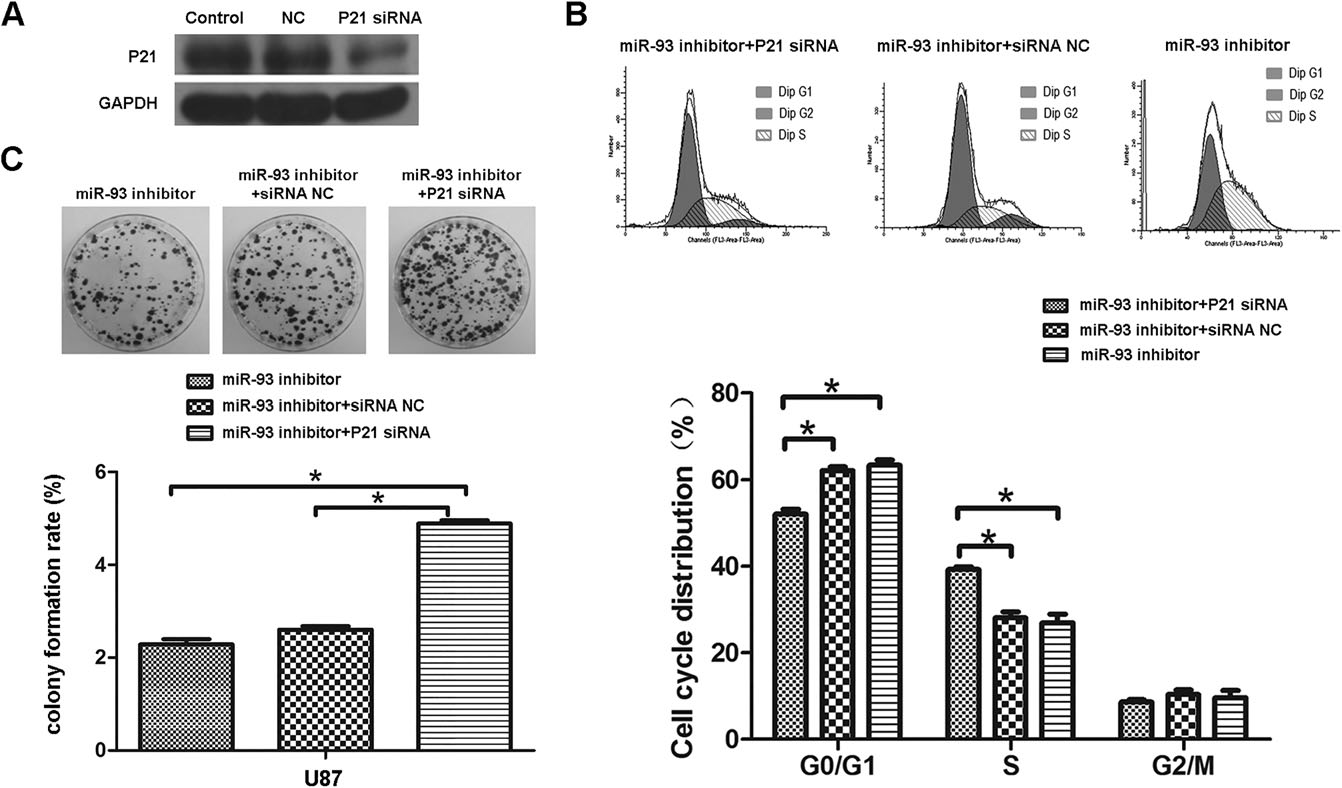
**Knockdown of p21 reverses miR-93 inhibitor mediated inhibition of cell proliferation.** (A) U87 cells were transfected with non-specific siRNA (NC) or P21 siRNA, respectively. Western blot was conducted to examine the protein expression of P21. (B) Cell cycle analysis was performed to examine the cell cycle distribution in each group, and quantification. (C) Colony formation assay was performed to examine the colony formation capacity in each group, and formation quantified. Data represented as mean±s.d; *P<0.05.

As miRNAs play a role via regulating their targets expression (Areeb et al., 2015), we further investigated the putative targets of miR-93 by using several common miRNA analysis software, including Pictar, MicroRNA.org, and Targetscan, and P21 (encoded by CDKN1A) was predicated to be a target gene of miR-93 (Fig. 4A). To verify this prediction, we constructed the wild type (WT) P21 3′UTR containing the putative binding sequences (GCACUUU) of miR-93 and the mutant type (MUT) P21 3′UTR, within which the binding sequences were changed into ‘AAAAAAA’. We then inserted them into the pMIR- REPORT miRNA Expression Reporter vector, generating WT P21-3′UTR plasmid and MUT P21-3′UTR plasmid, respectively. HEK293 cells were co-transfected with WT P21-3′UTR or MUT P21-3′UTR plasmid, and miR-NC or miR-93 mimic, respectively. After co-transfection for 48 h, dual-luciferase reporter assay was performed, and our data showed that the luciferase activity was significantly decreased in HEK293 cells co-transfected with WT P21-3′UTR plasmid and miR-93 mimic compared to the control group (Fig. 4B). However, it was unchanged in the other groups, when compared to the control group (Fig. 4B). These data indicate that P21 is a direct target gene of miR-93. We further examined the effects of miR-93 on the expression of P21 in U87 cells. Our data showed that knockdown of miR-93 enhanced the protein expression of P21 in U87 cells, while overexpression of miR-93 led to a significant decrease in the protein levels of P21 in SF127 cells, when compared to the control groups, respectively (Fig. 4C). Accordingly, we suggest that miR-93 can negatively mediate the protein expression of P21 by directly binding to its 3′UTR in glioma cell lines. In addition, we also examined several other cell cycle-related proteins, and found that knockdown of miR-93 increased the protein levels of P27 and P53, but decreased the Cyclin D1 protein levels in U87 cells, while overexpression of miR-93 reduced the protein levels of P27 and P53, but upregulated the Cyclin D1 protein levels in SF126 cells, when compared to the control groups, respectively (Fig. 4D). These data are consistent with the result of cell cycle analysis in U87 and SF126 cells after knockdown or overexpression of miR-93. We further studied whether P21 acted as a downstream effecter of miR-93 in glioma cells. P21 siRNA was used to transfect U87 cells. After transfection, the protein levels of P21 were significantly decreased compared to the control group, indicating that the P21 siRNA is effective (Fig. 5A). We further set three groups using U87 cells: miR-93 inhibitor, miR-93 inhibitor+siRNA NC, and miR-93 inhibitor+P21 siRNA. After that, we examined the cell cycle distribution in each group. As indicated in Fig. 5B, cells in the G0/G1 stage were markedly decreased in the miR-93 inhibitor+P21 siRNA, while cells in the S stage were significantly increased in the miR-93 inhibitor+P21 siRNA, when compared to the other two groups, respectively. These data suggest that knockdown of P21 reversed the suppressive effects of miR-93 inhibition on the cell cycle progression in U87 cells. We then examined the colony formation capacities in each group. As indicated in Fig. 5C, the colony formation rate was significantly higher in the miR-93 inhibitor+P21 siRNA group, when compared with that in the other two groups, respectively. These results suggest that P21, as a downstream effecter of miR-93, had suppressive effects on the colony formation capacities of U87 cells.

### Knockdown of miR-93 enhances the chemosensitivity of U87 cells to TMZ

**Fig. 6.**
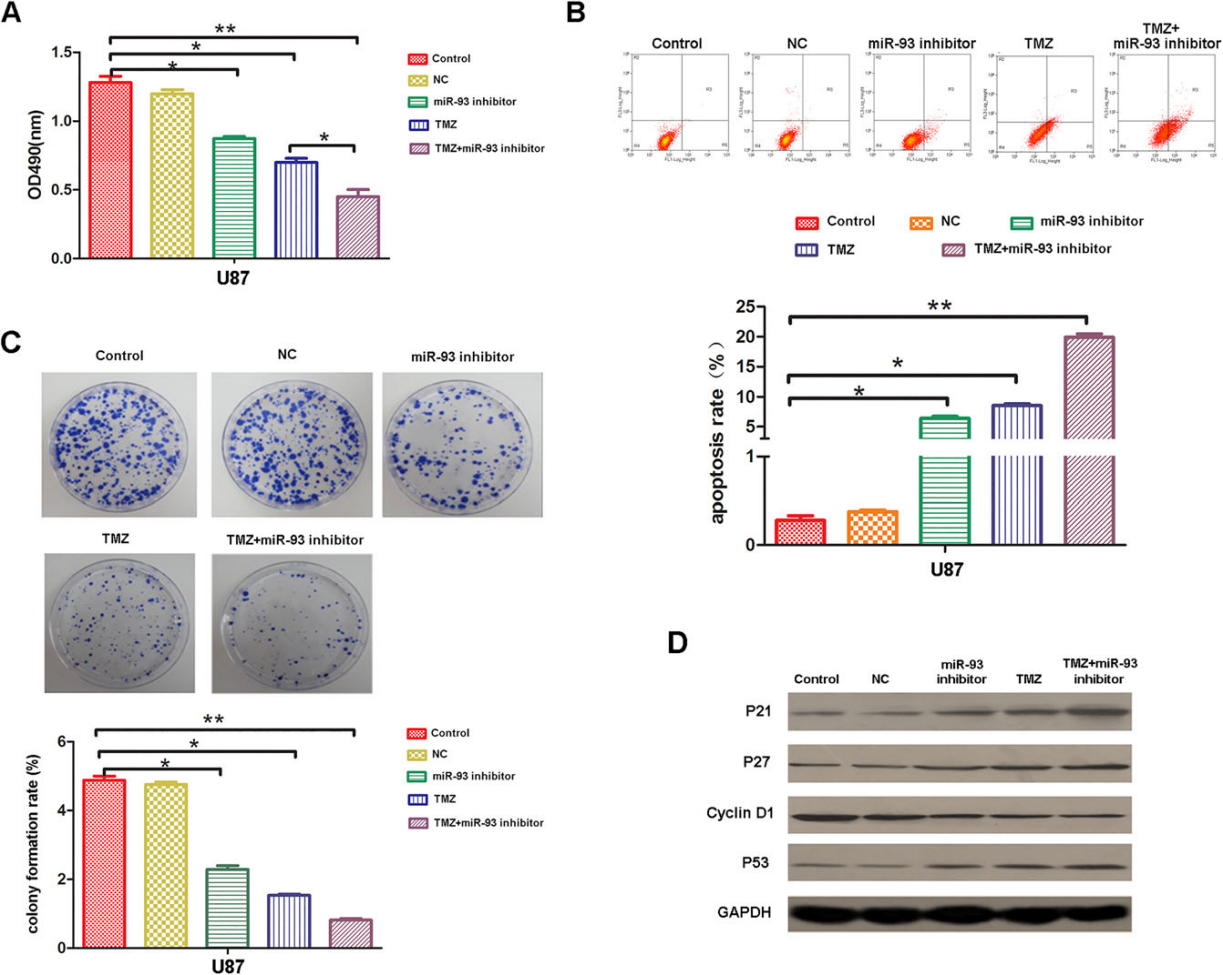
**Knockdown of miR-93 enhances the chemosensitivity of U87 cells to TMZ.** U87 cells were treated with miR-93 inhibitor or TMZ alone, or co-treated with both. Cells without any treatment or treated with miR-NC were used as mock or negative control. (A) MTT assay was used to measure the cell proliferation in each group. (B) Cell apoptosis assay was used to determine the cell apoptosis in each group. (C) Colony formation assay was used to evaluate colony formation capacity in each group. (D) Western blot was conducted to examine the protein expression of P21, P27, P53 and Cyclin D1 in each group. GAPDH was used as an internal reference. Data represented as mean±s.d; *P<0.05, **P<0.01, ***P<0.001.

We further studied the effects of miR-93 on the drug resistance in glioma cells. TMZ is the most common chemotherapy drug used in glioma. Therefore, we investigated whether inhibition of miR-93 could enhance the chemosensitivity of U87 cells to TMZ. TMZ (100 μmol/l) was used to treat U87 cells with or without transfection with miR-93 inhibitor for 24 h. MTT was used to examine the cell proliferation. As indicated in Fig. 6A, treatment with TMZ significantly inhibited U87 cell proliferation compared to the control group. Moreover, the cell proliferation was further decreased in the TMZ+miR-93 inhibitor group. Flow cytometry was further conducted to examine the cell apoptosis rate in each group. Our data showed that treatment with TMZ significantly induced cell apoptosis compared to the control group, and inhibition of miR-93 further enhanced the cell apoptosis in U87 cells treated with TMZ (Fig. 6B). After that, colony formation assay was conducted. We observed that TMZ inhibited the colony formation capacity of U87 cells, while knockdown of miR-21 further enhanced the suppressive effect of TMZ on colony formation of U87 cells (Fig. 6C). These above data indicate that inhibition of miR-93 could enhance the chemosensitivity of U87 cells to TMZ. After that, we examined the expression levels of cell cycle-related proteins in each group. We observed that knockdown of miR-93 promoted the effects of TMZ treatment on the protein levels of these cell cycle-related genes (Fig. 6D), suggesting that these proteins are involved in the miR-93-mediated chemosensitivity of U87 cells to TMZ.

## DISCUSSION

The detailed role and underlying mechanism of miR-93 in the regulation of glioma growth and chemoresistance remain largely unclear. In the present study, we observed that miR-93 was significantly upregulated in glioma, and increased miR-93 levels were significantly associated with the advanced malignancy. *In vitro* study showed that miR-93 could directly target P21, and promote the malignant phenotypes of glioma cells, as well as their chemoresistance to TMZ. Besides, several other cell cycle-related proteins including P27, P53 and Cyclin D1 were also mediated by miR-93 in glioma cells.

Recently, miRNAs have been found to play key roles in the development and progression of glioma, such as miR-23b (Chen et al., 2012a), miR-27b (Chen et al., 2011), miR-124 (An et al., 2013), and miR-203 (Dontula et al., 2013). In the present study, we used *in situ* hybridization and real-time RT-PCR to examined the expression of miR-93 in glioma, and found that it was significantly upregulated in glioma tissues compared to normal brain tissues. Moreover, we showed that its upregulation was significantly associated with the malignant progression as well as poor prognosis of glioma patients, suggesting that miR-93 may play an oncogenic role in glioma. Jiang et al. also reported that the expression of miR-93 was markedly upregulated in glioma tissues, and that the miR-93 levels were significantly correlated with clinicopathologic grade and overall survival in glioma, consistent with our findings (Jiang et al., 2015).

We further examined the miR-93 levels in several common glioma cell lines, and found that U87 cells showed the highest miR-93 levels, while SF126 cells showed the lowest miR-93 levels. To study the role of miR-93 in glioma cells, miR-93 inhibitor was used to decrease the miR-93 levels in U87 cells, while miR-93 mimic was used to increase its expression in SF126 cells. Further investigation showed that upregulation of miR-93 enhanced the proliferation, cell cycle progression, migration and invasion of SF126 cells, while knockdown of miR-93 suppressed these malignant phenotypes of U87 cells, suggesting that miR-93 may promote the growth and metastasis of glioma. Indeed, Fang et al. found that miR-93 could enhance the tumor growth of glioma cells *in vivo* (Fang et al., 2011). They showed that miR-93-oveexpressing glioma cells induced the formation of blood vessels in the tumor tissue, which in return facilitated cell survival, resulting in enhanced tumor growth (Fang et al., 2011). In addition, they identified integrin-β8 as a target of miR-93, and found that higher levels of integrin-β8 were associated with cell death in tumor mass and in human glioblastoma (Fang et al., 2011). In our study, we found that P21 was a direct target of miR-93, and its protein levels were negatively mediated by miR-93 in glioma cells. P21, also known as cyclin-dependent kinase inhibitor 1A, is a potent cyclin-dependent kinase inhibitor. It binds to and inhibits the activity of cyclin-CDK2 or -CDK4 complexes, and thus functions as a regulator of cell cycle progression at G1 (Wang et al., 2014; Agarwal, 2000). Moreover, P21 acts as a key regulator in S phase DNA replication and DNA damage repair through interacting with proliferating cell nuclear antigen (PCNA), a DNA polymerase accessory factor (Buscemi et al., 2014). In the present study, overexpression of miR-93 enhanced cell cycle progression, accompanied with decreased P21 levels in SF126 cells. On the contrary, knockdown of miR-93 induced a cell cycle arrest at G0/G1 stage, accompanied with an upregulation of P21 in U87 cells. Moreover, knockdown of P21 reversed the suppressive effects of miR-93 inhibition on the cell cycle progression in U87 cells. Based on these data, we suggest that the promoting effect of miR-93 on cell cycle progression as well as proliferation in glioma cells may be directly via inhibition of P21. Besides, the P21 expression has been found to be tightly controlled by the tumor suppressor P53 (Garner and Raj, 2008). In the present study, we found that miR-93 also had a suppressive effect on the protein levels of P53 in U87 and SF126 cells. Therefore, we suggest that miR-93 may not only directly inhibit the expression of P21 at the post-transcriptional level, but also through mediation of P53.

TMZ is a common chemotherapy drug used for the treatment of glioma (Zhang et al., 2015). In the present study, we for the firstly time reported that knockdown of miR-93 enhanced the chemosensitivity of glioma cells to TMZ, by inhibiting cell proliferation and colony formation and inducing cell apoptosis. Therefore, miR-93 may be a cause of chemoresistance of glioma to TMZ. Recently, some other miRNAs were also reported to be a regulator of chemoresistance in glioma. For instance, Chen et al. reported that knockdown of miR-221/222 sensitized glioma cells to TMZ by regulating apoptosis independently of p53 (Chen et al., 2012b). Shi et al. found that miR-125b conferred glioblastoma stem cells resistance to TMZ through mediation of apoptosis pathway (Shi et al., 2012). Accordingly, our study expands the understanding of miRNA in the regulation of chemoresistance in glioma. In addition, we found that knockdown of miR-93 promoted the suppressive effects of TMZ treatment on the protein levels of P21, P27 and P53, as well as the promoting effects on Cyclin D1. We speculated that P21, as a direct target of miR-93, might be involved in the miR-93-mediated chemoresistance of glioma cells to TMZ, which should be verified in the future studies.

In conclusion, our study reveals an oncogenic role of miR-93 in the regulation of proliferation, cell cycle progression, colony formation, migration, invasion, and chemoresistance in glioma cells, possibly via directly targeting P21.

## MATERIALS AND METHODS

### Tissue specimen collection

The present study was approved by the Ethics Committee of Xiangya Hospital of Central South University, Changsha, China. A total of 43 cases of glioma tissues as well as eight cases of normal brain tissues were collected at the Department of Neurosurgery, Xiangya Hospital from 2012 to 2014. Among these glioma patients, 27 are male, 5-71 years old with an average 38.3±21.4; and 16 are female, 8-67 years old with an average 35.4±19.7. In addition, there are six cases of WHO I, 20 cases of WHO II, 11 cases of WHO III, and six cases of WHO IV. All written informed consents have been obtained. The histomorphology was confirmed by the Department of Pathology, Xiangya Hospital. Tissues were immediately snap-frozen and stored in liquid nitrogen after surgical removal.

### *In situ* hybridization

Slides were deparaffinized and dehydrated with xylene for 10 min, and then put through an ethanol gradient (100%, 95%, 90%, 80% and 70%, each for 5 min), and rinsed with dH2O. The slides then were treated with 3% H2O2 for 10 min and washed three times by dH2O. The slides were then treated with pepsin solution for 10 min at 37°C, and washed three times with 0.5 M PBS for 5 min and once with dH2O for 10 min at room temperature. Following 3 h incubation with pre-hybridization solution at 37°C, slides were incubated with miR-93 probe (2 μg/ml, Exiqon, Denmark) overnight at 55°C. Then, slides were incubated in 2× saline sodium citrate (SSC) for 30 min at 37°C, and then washed with 0.5× SSC for 15 min, and then washed with 0.2× SSC for 15 min. Following 30 min blockade with normal goat serum at 37°C, slices were incubated with biotin anti-digoxin IgG for 90 min at 37°C and washed three times with 0.5 M PBS for 5 min, followed by Strept Avidin-Biotin-Complex and biotin-peroxidase incubation for 30 min at 37°C. After washing in 0.5 M PBS for 20 min, the slides were visualized with 3,3′-diaminobenzidine (DAB) (Maixin, Fujian, China) for 5 min and counterstained with haematoxylin for 90 s. The slides were mounted and dried, and photographed using an EVOS inverted fluorescent microscope (EVOS, USA). The expression of miR-93 in ISH was evaluated as others described (Luo et al., 2015) by two independent pathologists. Briefly, the miR-93 staining intensity was scored as 0 (negative), 1(+), 2(++), and 3 (+++). The extent of staining was scored as 0∼1.0 (0%∼100%). The final staining score (0-3) was calculated as the multiplication of the intensity score and extent score. The final score ≥1 was defined as high expression, otherwise was defined as low expression.

### Cell culture

Human glioma cell lines U87, SF126, SF767, A172 and SHG44 were purchased from the American Type Culture Collection, ATCC, USA. HEK293 cells and human glioma cell line U251 was purchased from the Cell Bank of Chinese Academy of Sciences (Shanghai, China). SF126 and SF767 were human glioma cell lines. SHG44 was WHO II human astroglioma cell line. U87, A172 and U251 were WHO IV human glioblastoma cell lines. Cells were cultured in RPMI1640 (Life Technologies, USA) added with 10% fetal bovine serum (FBS, Hyclon, USA) at 37°C in a humidified atmosphere with 5% CO_2_.

### Real-time RT-PCR assay

Total RNA of tissues or cells was extracted using Trizol Reagent (Life Technologies), according to the manufacture's instruction. Reverse Transcription Kit (Life Technologies) was used to convert RNA into cDNA, according to the manufacture's instruction. For miRNA detection, real-time PCR was conducted by using a SYBR Green-containing PCR (Life Technologies) on ABI 7500 thermocycler. U6 gene was used as an internal reference. The primers for miR-93 and U6 were purchased from GeneCopoeia, USA. The PCR reaction conditions were 95°C for 3 min, followed by 40 cycles of 95°C for 12 s and 62°C for 30 s. The relative expression was analyzed by the 2^−ΔΔCt^ method (Agarwal, 2000).

### Transfection

Lipofectamine 2000 (Life Technologies) was used to perform transfection, in accordance with the manufacturer's instruction. MiR-negative control (miR-NC, GenePharma, Shanghai, China), miR-93 mimic (GenePharma), miR-93 inhibitor (GenePharma), inhibitor-NC (GenePharma), p21 siRNA, p21-NC, or Lipofectamine 2000 was diluted with OPTI-MEM (Life Technologies), respectively. The diluted Lipofectamine 2000 was added into the diluted plasmid, miR, or siRNA, respectively. After incubation at room temperature for 20 min, the above mixture was added into the cell suspension, respectively, which was then incubated at 37°C, 5% CO_2_ for 6 h. After that, the transfection mixture was replaced by DMEM with 10% FBS. Cells were then cultured for 48 h before the following assays.

### Western blotting

Cells were lysed in the protein lysis buffer (Xinyu Biotechnology, Shanghai, China). The protein concentration was determined using the BCA Protein Assay Kit (Pierce Chemical, Rockford, IL, USA). Protein was separated with 10% SDS-PAGE, transferred to a PVDF membrane (Millipore), and then blocked in 5% nonfat dried milk in TBST for 2 h. The PVDF membrane was then incubated with primary antibodies (Rabbit monoclonal anti-CyclinD1, cat no. ab137875, dilution: 1:1000; Rabbit monoclonal anti-P21, cat no. ab109520, dilution: 1:500; Rabbit monoclonal anti-P27, cat no. ab92741, dilution: 1:500; Rabbit polyclonal anti-P53, cat no. ab31333, dilution: 1:200; and mouse monoclonal anti-GAPDH, cat no. ab8245, dilution: 1:3000. All the antibodies were from Abcam (Cambridge, UK)) at 4°C overnight, and then washed with TBST four times. Then, the PVDF membrane was incubated with mouse anti-rabbit secondary antibody for 1 h at room temperature, and then washed with TBST four times. All antibodies were purchased from Thermo Fisher, USA. The immune complexes were then detected using the ECL Western Blotting Kit (Pierce) and X-film (Kodak, Tokyo, Japan). ImageJ software (NIH) was used to analyze the relative protein expression, represented as the density ratio versus GAPDH.

### Cell proliferation detection

MTT assay was used to examine the cell proliferation. Cells (2×10^3^) in each group were plated into a 96-well plate and cultured for 12, 24, 48, or 72 h at 37°C with 5% CO_2_. After that, 20 μl of MTT (5 mg/ml, Sigma, USA) was added. After incubation at 37°C for 4 h, 150 μl of DMSO was added. After incubation at room temperature for 10 min, the formazan production was detected by determining the optical density (OD) at 490 nm using Elx800 enzyme immunoassay analyzer (Bio-Tec, VT, USA).

### Colony formation assay

Colony formation rate was measured by plate colony formation assay. About 400 cells were added to each well of a 6-well plate. Cells were cultured at 37°C for 2 weeks. After that, cells were gently washed and fixed with 4% paraformaldehyde (Sigma) for 20 min. Then, cells were stained with 0.1% crystal violet (Sigma) for 30 min, and washed and dried in the air. Viable colonies containing at least 10 cells were counted.

### Wound healing assay

Wound healing assay was conducted to examine the migratory capacity of U87 cells. Cells were seeded into 35-mm dishes pre-coated with fibronetin, and cultured to 100% confluence. Then, a scratch was made by using a sterile tip. Then, cells were cultured for 24 h, and observed under microscope. The width of wounds was determined at 0 h and 24 h.

### Cell invasion assay

Cell invasion assay was performed using transwell chambers (BD, USA), which had been pre-coated with Matrigel. Cell suspension (10^5^ cell/ml) was prepared in serum-free media, and 300 μl of cell suspension was added into the upper chamber. Then, 500 μl of DMEM with 10% FBS was added into the lower chamber. After cultured for 24 h, the filters were fixed in 10% formaldehyde and stained by 0.1% crystal violet for 20 min. Then, a cotton-tipped swab was used to wipe out the cells that did not invade through the pores. Then, invasive cells were observed under EVOS inverted fluorescent microscope (EVOS, USA).

### Cell cycle analysis

Flow cytometry was performed to determine the cell cycle in all groups. Cells in each group were resuspended in 70% ethanol. After fixed overnight at −20°C, cells were pelleted, washed twice in 1× PBS with 3% BSA, and pelleted. After that, cells were resuspended and incubated for 30 min at room temperature in propidium iodide (PI) staining buffer containing 3% BSA, 40 µg/ml propidium iodide, and 0.2 mg/ml RNase in 1× PBS. DNA content analyses were carried out using the flow cytometry (FACSCalibur, Beckman Coulter).

### Cell apoptosis analysis

The cell apoptotic levels were examined using the Annexin V-fluorescein isothiocyanate (FITC) apoptosis detection kit (BD Pharmingen, San Diego, CA, USA), according to the manufacturer's instruction. Cells in each group were re-suspended in 1× binding buffer solution with Annexin V-FITC and PI and incubated for 15 min at room temperature in the dark. The apoptotic rate was determined using EPICS ALTRA flow cytometry (Beckman, USA).

### Bioinformatic prediction

Pictar, MicroRNA.org, and Targetscan were used to analyze the putative target genes of miR-93.

### Dual luciferase reporter assays

The wild type (WT) of P21 3′-UTR was constructed by PCR and inserted into the pMIR- REPORT miRNA Expression Reporter vector (Ambion, USA). The mutant type (MUT) of P21 3′-UTR was constructed by using Easy Mutagenesis System kit (Promega, Madison, WI, USA), in accordance with the manufacture's protocol, and then inserted into the pMIR- REPORT miRNA Expression Reporter vector. HEK293 cells were co-transfected with WT P21-3′UTR or MUT P21-3′UTR plasmid (400 ng), and miR-NC or miR-93 mimic (50 nM), using Lipofectamine 2000. After co-transfection for 48 h, the dual-luciferase reporter assay system (Promega) was used to determine the activities of *Renilla* luciferase and firefly luciferase. The *Renilla* luciferase activity was normalized to the firefly luciferase activity.

### TMZ treatment

Temozolomide was used to treat U87 cells with or without transfection with miR-93 inhibitor. After treatment for 3 h, cell proliferation and apoptosis as well as the protein levels of cell cycle-related genes were examined.

### Statistical analysis

Results showed in figures are expressed as mean±s.d. Analysis of data was performed by using SPSS 17.0 software (IBM). Student *t*-tests, one-way ANOVA or chi-square test was used to analyze the significance of differences among groups depending on the experimental conditions. Statistics significance was evaluated by *P* values of <0.05.
